# *Plasmodium falciparum* VAR2CSA-Specific IgG Subclass Responses Reflect Protection Against Low Birth Weight and Pregnancy-Associated Malaria

**DOI:** 10.3389/fimmu.2021.610305

**Published:** 2021-04-21

**Authors:** Bernard Tornyigah, Tania d’Almeida, Guillaume Escriou, Firmine Viwami, Nadine Fievet, Adrian J. F. Luty, Achille Massougbodji, Morten A. Nielsen, Philippe Deloron, Nicaise Tuikue Ndam

**Affiliations:** ^1^ Université de Paris, MERIT, IRD, Paris, France; ^2^ Department of Immunology, Noguchi Memorial Institute for Medical Research, College of Health Sciences, University of Ghana, Legon, Ghana; ^3^ Université Pierre et Marie Curie, Ecole doctorale 393 Pierre Louis de Santé publique, Paris, France; ^4^ Centre d’Etude et de Recherche sur le Paludisme Associé à la Grossesse et à l’Enfance, Cotonou, Benin; ^5^ Centre for Medical Parasitology, University of Copenhagen, Copenhagen, Denmark

**Keywords:** pregnancy, malaria, VAR2CSA, IgG subclasses, IgG3, IgG4

## Abstract

Sequestration of *Plasmodium falciparum*-infected erythrocytes expressing the VAR2CSA antigen in the placenta results in poor pregnancy outcomes, including low birth weight and maternal anemia. Antigen-specific antibody-mediated immunity is acquired during successive pregnancies. Thus, evaluating VAR2CSA-specific IgG profiles among pregnant women will increase knowledge on the immunological mechanisms associated with protection, and help in the development of VAR2CSA-based placental malaria vaccines. Using the PAMVAC candidate vaccine antigen, we assessed anti-VAR2CSA IgG subclass responses of a cohort of pregnant Beninese, and analyzed their relationships with pregnancy outcomes. Cytophilic IgG1 and IgG3 responses were the most frequent, with prevalences ranging from 28% (IgG3) up to 50% (IgG1). Elevated levels of VAR2CSA-specific total IgG and cytophilic IgG3 during pregnancy were consistently associated with higher birth weights, whilst high levels of IgG4 were associated with a reduced risk of placental infections. This suggests that protective anti-VAR2CSA IgG responses are coordinated between both cytophilic and non-cytophilic antibodies.

## Introduction

*Plasmodium falciparum* malaria continues to be a significant human disease burden throughout the tropics. In sub-Saharan Africa, placental malaria (PM) is known to cause low birth weight and maternal anemia (1, 2). The adverse effects associated with PM are due to the sequestration of infected erythrocytes (IEs) in the placental intervillous spaces. The often massive accumulation of IEs is mediated by VAR2CSA, a parasite ligand that promotes adhesion of IEs to placental chondroitin sulfate A (CSA) (3–5). This pathophysiological feature, which led to the recognition of placental malaria as a specific syndrome, is particularly frequent in first pregnancies in areas where *P. falciparum* malaria is highly endemic (6). Women who have had PM develop anti-VAR2CSA IgG antibodies that protect them from the adverse effects of PM during subsequent pregnancies, making this protein an attractive target for vaccine development (4, 7). Anti-VAR2CSA IgGs have been shown to inhibit the binding of VAR2CSA-expressing infected erythrocytes to CSA (8–10). Further studies identified the Id1-DBL2X-Id2 region, in the N-terminal part of VAR2CSA, as being the minimal binding domain mediating binding between VAR2CSA and placental CSA (9), thus making it appropriate for the development of a VAR2CSA-based vaccine with an antigenic construct easier to produce in recombinant form. A Phase 1 clinical trial has recently been completed with such an antigenic construct (10).

Our team has already demonstrated in a longitudinal study that high levels of IgG with specificity for the N-terminal part of VAR2CSA present in women early in pregnancy were associated with better pregnancy outcomes (11). Additionally, a recent systematic review suggests that IgG levels against VAR2CSA antibodies are likely markers of infection rather than correlates of protection (12). However, information is lacking on which of the IgG subclasses targeting the vaccine construct are responsible for protective immunity and also on the dynamics of such protective antibody levels during pregnancy. Here, we used samples from a group of women whose pregnancy outcomes were well characterized in the Strategies to Prevent Pregnancy Associated Malaria (STOPPAM) study in Benin and from whom consecutive blood samples and clinical data were available from the time of inclusion until delivery. Using measurements of naturally acquired antibody targeting the PAMVAC vaccine Id1-Id2a recombinant antigen on consecutive plasma samples, we analyzed the link between IgG subclasses with different pathological outcomes of pregnancy.

## Methods

### Study Site and Population

The current study made use of samples collected from a subgroup of women drawn from the STOPPAM project initially designed to characterize the pathology of placental malaria, to determine the factors important for optimal use of intermittent preventive treatment of malaria in pregnancy (IPTp) and for vaccine development against pregnancy-associated malaria. A detailed description of the STOPPAM study design has been reported (13). For the current work, 470 women were included based on their pregnancy outcome as previously reported (14). This sub-group included women who had placental infection at delivery, were anemic, whose infants had low birth weight, in whom intrauterine growth retardation was identified based on fetal growth curves derived from ultrasounds to define children born small-for-gestational-age (SGA), or whose infants were born prematurely. Another group of pregnant women from the same area with no pathological outcome at delivery was also included. Briefly, babies were classified as premature if their gestational age, assessed by ultrasound before 24 weeks of gestation, was <37 weeks at delivery. Fetal growth restriction was approximated by low birth weight for gestational age. A baby was defined as SGA if the birth weight was below the 10^th^ percentile of fetal weight for the gestational age (15). Maternal anemia at delivery was defined as a hemoglobin level below 10 g/dl. Placental malaria was defined as a positive placental blood smear (16). At inclusion and each antenatal or emergency visit, a thick blood smear was processed for malaria diagnosis. Venous blood was collected, and plasma samples were separated and frozen at −80°C for further use. All women diagnosed with *P. falciparum* infection by thick blood smear during the follow up were treated with quinine according to national guidelines applicable at the time of the study.

### Ethical Statement

The STOPPAM study obtained ethical approval from two independent institutional ethics committees: the Comité Consultatif de Déontologie et d’Éthique of IRD in France and the Comité d’Éthique de la Faculté des Science de la Santé, Université d’Abomey Calavi, in Benin. All participants provided written consent.

### Total IgG and Subclasses Quantification

To measure antibody response against VAR2CSA, we used the PAMVAC vaccine candidate, a recombinant protein representing the ID1-ID2a construct of VAR2CSA from the FCR3 variant of *P. falciparum* (10, 17). Antibody levels with specificity for the ID1-ID2a recombinant protein were determined by a previously described indirect ELISA protocol (7, 10). In brief, antigen-coated (0.5 µg/mL) and bovine serum albumin (BSA)-blocked 96-well ELISA plates (Maxisorp, NUNC, Denmark) were incubated overnight at 4°C with 100 μl/well of test plasma samples in duplicate at dilutions optimized for each subclass (1:200 for total IgG; 1:100 for IgG1 and IgG2 and 1:50 for IgG3 and IgG4) in phosphate-buffered saline (PBS) with 2% BSA. Pools of highly reactive plasma from exposed pregnant Beninese previously characterized on recombinant full-length ectodomain of VAR2CSA (FV2) (11), and non-immune plasma from unexposed French volunteers, were used on each plate as internal controls. Reactions were developed with goat anti-human IgG conjugated to horseradish peroxidase (SIGMA, France) followed by the substrate TMB (SIGMA, France). The color reaction was stopped by the addition of 0.2M H_2_SO_4_ and optical densities (ODs) were read at 450 nm. In between all incubation steps, plates were washed 3 times with PBS, pH 7.4, containing 0.1% Tween 20, using an automated plate washer.

ELISA for the subclasses followed the same procedure except using mouse anti-human IgG subclasses Fc secondary antibody conjugated to HRP (MH1715, MH1722, MH1732, or MH1742, all from Invitrogen, France). IgG4 antibody levels were also measure against ICAM1 binding DBL-β domain from IT4 VAR13 *Pf*EMP1 (18) donated by Professor Jensen. All reagents were used at predetermined concentrations and optical densities (OD) generated from the antibody measurements were converted to arbitrary units (AUs), as described (19). Briefly, AUs were calculated as follows:

$$AU=100*\frac{\text{ln}(0D\;test\;sample)-\text{ln}(0D\;negative\;sample)}{\text{ln}(0D\;positive\;sample)-\text{ln}(0D\;negative\;sample)}$$

### Statistical Analysis

For each measured IgG subtype, the mean ODs from non-immune plasma from each plate were calculated plus 2 standard deviations to define a common cut-off value for each IgG isotype above which samples were deemed antibody-positive. Seroprevalence was calculated as the proportion of samples with OD above this cut-off. The outcome variable was the level of IgG antibodies in peripheral maternal blood. Five principal outcome variables were created: the level of total IgG, the levels of IgG1, IgG2, IgG3, and IgG4. As multiple antibody measurements (inclusion, antenatal visits, and delivery) were available, appropriate hierarchical mixed models were used for univariate and multivariate analyses, performed with Stata^®^ software, Version 12 (StatCorp LP, College Station, TX, USA). The level of antibodies was tested in relation to the following different covariates: maternal age, gravidity, malaria at inclusion, number of peripheral malaria infections identified until the date of plasma collection, parasite density, and at delivery according to pregnancy outcome including anemia and placental infection in mothers; low birth weight, prematurity, intrauterine growth retardation in newborns.

All factors with a *p*-value <0.20 during univariate analyses were included in the multivariate step. A backward stepwise procedure was used to select factors of the final model. Statistical significance was set at *p*<0.05.

## Results

The STOPPAM cohort subgroup used in this work comprises Beninese pregnant women (mean age, 26 years) followed up during the course of pregnancy. Among the 470 women included in this study, 11 (2.3%) attended 3 antenatal visits (ANV) while 415 (88.3%) attended all the 5 scheduled visits (inclusion, ANV1, ANV2, ANV3, and delivery). Amongst the study cohort 78 (16.6%) were primigravidae, 63 (13.4%) had evidence of placental malaria at delivery, and 82 (17.4%) were anemic at delivery. Forty-nine (10.1%) women gave birth to infants with LBW (<2500g), 53 (11.3%) with SGA, 24 (5.1%) women gave birth prematurely (before 37 gestational weeks), and 272 women had no pathological outcomes. The characteristics of women who had their IgG profiles determined are presented in **Table 1**.

**Table 1:** Characteristics of the population.

Mothers	N=470
**Maternal age (years)**	26.48± 6.18
**Primigravidae**	78 (16.6%)
**Multigravidae**	392 (83.4%)
**Malaria infection during the follow-up (BS)**	
0	234 (49.8%)
1	132 (28.1%)
2	63 (13.4%)
≥3	41 (8.7%)
**Placental malaria**	
Yes	63 (13.4%)
No	405 (86.2%)
**Unknown**	2 (0.4%)
**Maternal anemia at delivery**	
Yes	82 (17.4%)
No	358 (76.2%)
Unknown	30 (6.4%)
**Malaria at inclusion (BS and PCR)**	
Negative	265 (56.4%)
Submicroscopic	121 (25.74%)
BS Positive	80 (17.0%)
**Newborns**	
**Birth weight (grams)**	2982.22± 434.23
**Low birth weight**	
Yes	49 (10.1%)
No	418 (88.9%)
Unknown	3 (0.6%)
**SGA**	
Yes	53 (11.3%)
No	417 (88.7%)
**Prematurity**	
Yes	24 (5.1%)
No	446 (94.9%)

BS, Blood smear; SGA, small for gestational age.

### Antibody Profiling and Factors Associated

**Figure 1:**
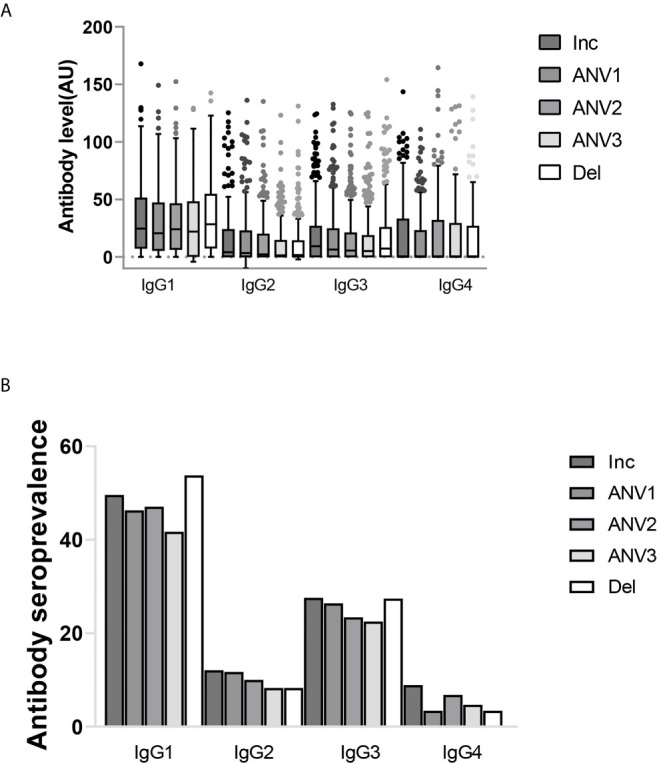
IgG subclass profile to ID1-ID2a recombinant protein from pregnant women followed during the course of pregnancy (inclusion, antenatal visits (ANV) and delivery). **(A)** The distribution of IgG subclasses. Shown are IgG subclasses antibody levels (median and IQR) in arbitrary units. **(B)** IgG subclass seroprevalence. The profile of IgG subclasses clearly shows a cytophilic IgG’s (IgG1 and IgG3) are the predominant IgGs while low prevalence of non-cytophilic IgG’s (IgG2 and IgG4) were also observed.

The distribution of IgG subclass responses shows that cytophilic IgG1 and IgG3 were measured in a higher number of women compared to IgG2 and IgG4 (**Figure 1A**). Analysis of the seroprevalence of the different IgG subclasses shows that IgG1 and IgG3 were the most prevalent with rates ranging from 49.6% to 53.8% for IgG1 and from 27.6% to 27.4% for IgG3 according to the time of pregnancy. IgG2 and IgG4 were less prevalent with rates ranging from 8.3% to 12.1% and from 3.4% to 8.9%, respectively (**Figure 1B**). The ranges were larger for subclass measurements compared to total IgG leading to larger dispersion of values (**Figure 1**). When taking into account repeated measurements made on consecutive samples of the same woman in the analyses, plasma levels of IgG (both total and subclasses) gradually decreased over the course of pregnancy, except for IgG4 which was consistently low throughout. (**Table 2**, **Figure 1A**).

**Table 2:** Relationship between antibody levels to ID1-ID2a construct of VAR2CSA and pregnancy outcomes (Total IgG and IgG1).

Covariates	Total IgG						IgG 1					
	Univariate			Multivariate^(**)^			Univariate			Multivariate^(**)^		
	β	95% CI	*p*	Adj β	95% CI	*p*	β	95% CI	*p*	Adj β	95% CI	*p*
**Primiparity** No	_			_			_					
Yes	-9.07	-14,94; -3,21	**0.002**	-9.9	-15,91; -3,90	**0.001**	-3.29	-12,68; 6,10	0.49			
Malaria Infections^(*)^ 0	_			_			_			_		
1	5.18	3,62; 6,75		4.46	2,83; 6,09		6.16	3,24; 9,7	**<10^-3^**	4.92		**<10^3^**
2	7.73	3,75; 11,70	**<10^-3^**	7.93	3,83; 12,02	**<10^-3^**	12.92	4,50; 21,34		12.37		
≥3	13.56	3,13; 23,99		14.30	3,92; 24,68		7.66	-9,20; 24,52		5.44		
**Parasite density**	0.0002	0,00008; 0,0002	**<10^-3^**	0.0001	0,000016; 0,00027	**0.028**	0.0004	0,0002; 0,0005	**<10^-3^**	0,00002	-0,0003; 0,0003	0,89
**Prematurity** No	_						_					
Yes	-6.93	-16,93; 3,08	0.17	-4,04	-14,45; 6,38	0,45	0.05	-13,75; 13,84	0.99	-0,84	-18,01; 16;33	0,93
**SGA** No	_						_			_		
Yes	-4.45	-11,48; 2,58	0.22	-4,04	-12,04; 3,96	0,32	1.47	-9,39; 12,35	0.79	-1,58	-13,65; 10,48	0,80
**Placental malaria** No	_			_			_			_		
Yes	3.69	-2,77; 10,14	0.26	1,73	-4,70; 8,16	0,59	11.43	-0,88; 23,73	0.07	7,88	-4,65; 20,41	0,22
**LBW** No	_						_			_		
Yes	-9.04	-16,22; -1,85	**0.014**	-10.74	-18,04; -3,45	**0.004**	-0,59	-11,48; 10,30	0.92	-6,24	-17,33; 4,86	0,27
**Maternal anemia** No	_						_			_		
Yes	1,74	-3,99; 7,48	0.55	0,44	-5,18; 6,06	0,88	3.31	-5,87; 12,49	0.48	1,65	-7,52; 10,82	0,73

^(*)^ number of documented blood smear positive during follow-up.

^(**)^ Multivariate analysis were adjusted for timepoint.

When comparing levels by gravidity, it was clear that total IgG levels remained consistently higher in multigravid women compared to primigravidae, in both univariate and multivariate analysis. However, this observation was not so clear for individual IgG subclasses. When taking into account infections that were documented during the follow-up in relation to the time of antibody measurement, a high level of all subclasses were consistently observed in infected women, even though this reached significance only for IgG1 and IgG3. A similar observation was made for antibody subclass levels and parasite density of the infections documented during follow-up, but statistical significance in multivariate analysis was reached only for total IgG (**Table 2**) (P = 0.028) and IgG2 (P = 0.03).

### Relationship With Pregnancy Outcomes

To compare the level of IgG during pregnancy in women with well-defined pregnancy outcomes, we performed a hierarchical mixed model analysis and factors with p-value < 0.2 were further incorporated into the final multivariate model (**Table 3**). For total IgG, low levels of antibodies were consistently observed throughout the follow-up in women who delivered babies with low birth weight in univariate analysis (P = 0.014). This observation was further confirmed in the multivariate model (P = 0.004). However, for the other adverse pregnancy outcomes, no significant difference in the antibody levels of total IgG with specificity for the ID1-ID2a PAMVAC antigen was observed. We then tested in a similar way the antibody levels of the IgG subclasses. For IgG1 and IgG2, no significant differences were observed with the different pathological markers at delivery. Levels of IgG3 antibodies were consistently low in women who delivered low birth weight babies compared to those who delivered babies with normal birth weight (multivariate analysis, P = 0.01). Throughout pregnancy, IgG4 antibody levels were low in women presenting with placental infection at delivery compared to women without parasites in their placenta (multivariate analysis, P = 0.03). However, there was no association between IgG4 and placenta infection in data obtained in the same way using a comparable construct from a ICAM1 binding DBL-β domain from IT4 VAR13 *Pf*EMP1 which is not implicated in PM (β=-0.004; p-value = 0.33). Apart from LBW and placental malaria, we did not observe an association between the other pregnancy outcomes and antibody levels measured.

**Table 3:** Relationship between antibody levels to ID1-ID2a construct of VAR2CSA and pregnancy outcomes (IgG2, IgG3 and IgG4).

Covariates	IgG 2						IgG 3						IgG 4					
	Univariate			Multivariate^(**)^			Univariate			Multivariate^(**)^			Univariate			Multivariate^(**)^		
	β	95% CI	*p*	Adj β	95% CI	*p*	β	95% CI	*p*	Adj β	95% CI	*p*	β	95% CI	*p*	Adj β	95% CI	*p*
**Primiparity** No	_						_						_					
Yes	-7.15	-15,52; 1,23	0.09	-7,32	-15,69; 1,05	0,09	2.17	-4,89; 9,23	0.55				-0.08	-6,32; 6,16	0.98			
Malaria Infections^(*)^ 0	_						_			_			_					
1	2.21	0,09; 4,32	0.23				8.12	5,47; 10,77	**<10^-3^**	7.94	5,21; 10,66	**<10^-3^**	-1.81	-6,57; 2,96	0.7			
2	0.93	-4,78; 6,64					10.08	2,46; 17,69		10.12	2,47; 17,76		-1.54	-15,43; 12,36				
≥3	-0.35	-14,35; 13,64					2.97	-12,26; 18,22		2.93	-12,37; 18,22		-13.56	-41,33; 14,20				
**Parasite density**	0.0001	-0,0001; 0,0002	0.09	0.0001		**0.03**	0.00004	-0,0001; 0,0002	0,59				-0.0001	-0,0004; 0,0001	0.33			
**Prematurity** No	_			_			_			_			_			_		
Yes	-2.9	-14,13; 8,33	0.61	-3,49	-15,17; 8,19	0,56	-0.46	-10,75; 9,82	0.92	9,29	-3,39; 21,98	0,15	-1.46	-10,54; 7,62	0.75	-0,26	-11,61; 11,09	0,96
**SGA** No	_						_						_					
Yes	0.25	-9,10; 9,60	0.96	-1,48	-11,73; 8,77	0,78	1.87	-6,36; 10,09	0.66	6,62	-2,38; 15,61	0,15	-4.39	-11,54; 2,76	0,23	-3,33	-10,56; 3,89	0,36
**Placental malaria ** No	_			_			_			_			_					
Yes	8.99	-1,89; 19,88	0.10	10,09	-1,20; 21,39	0,08	-1.06	-10,17; 8,05	0.82	-2,18	-11,47; 7,11	0,65	-8.86	-16,97; -0,75	**0.03**	-8.86	-16,97; -0,75	**0.03**
**LBW** No	_			_			_			_			_			_		
Yes	-1.01	-10,40; 8,38	0.83	-0,91	-14,75; 12,94	0,89	-5.58	-13,69; 2,52	0,18	-10.79	-19,15; -2,45	**0.01**	-4,15	-11,32; 3,01	0,26	-2,49	-10,23; 5,23	0,53
**Maternal anemia** No	_			_			_			_			_			_		
Yes	-2.27	-10,03; 5,50	0.56	-2,04	-10,27; 6,18	0,63	1.04	-5,95; 8,03	0.77	-0,64	-7,64; 6,36	0,86	-0.92	-7,09; 5,25	0.77	-0,91	-7,27; 5,44	0,77

^(*)^ number of documented blood smear positive during follow-up.

^(**)^ Multivariate analysis were adjusted for timepoint.

## Discussion

In this study of a cohort of pregnant Beninese, we determined the levels of total IgG and of IgG subclasses with specificity for the PAMVAC vaccine antigen. We focused on the PAMVAC antigen currently undergoing vaccine development to decipher the specific antibody response that women develop when exposed to placental-type parasites. The levels of total IgG with specificity for the PAMVAC antigen were consistently lower in women giving birth to low birth weight babies, unlike other women, regardless of the gestational age at which the plasma sample was taken. These results confirmed our initial observations that low levels of total IgG directed to recombinant DBL1X-Id1-DBL2X of VAR2CSA in early pregnancy were associated with an increased risk of low birth weight (11). The current study emphasizes the fact that this particular association with risk of low birth weight persists throughout pregnancy. Moreover, it should be noted that, as was expected, there were relationships between the antibody levels measured here and the level of exposure to malarial infections or the gravidity status of the women, further supporting the relevance of the observations. Importantly, a recent systematic review by Cutts and his colleagues (12) by analyzing antibody data, most of which was obtained at delivery suggests that IgG levels against VAR2CSA antigen are “likely markers of infection rather than correlates of protection”. These observations are in line with those of this work which analyzed antibody levels during pregnancy on a longitudinal angle. This allowed underline the antigenic properties which can differ between different domains or epitopes of an antigen and thus provides additional justification for assessing whether VAR2CSA antibody subclass might better explain the discrepancies between studies.

One noteworthy observation is the association between low levels of cytophilic IgG3 antibody with low birth weight, although IgG1 was the predominant antibody subclass. This finding is similar to that of a study in Senegal (19) and suggests cytophilic IgG3 is the main effector in the prevention of LBW, reflecting the observation with total IgG. From a mechanistic point of view, IgG3 could be acting through either a direct inhibition of infected erythrocyte adhesion to CSA in the placenta, or an ability to mediate opsono-phagocytosis by effector cells, or through sensitization and activation of natural killer cells and the complement system (20–22). As LBW is among the most frequent detrimental outcomes of PM, this is an indication that any vaccine for PM should be demonstrably able to elicit IgG3.

Interestingly, we also observed that higher levels of IgG4 were associated with a reduced risk of placental infection. The IgG4 subclass, unlike IgG3, does not activate complement or act as an opsonin, suggesting that the role of such antibodies in any protective mechanism could only be achieved through the inhibition of infected erythrocyte adhesion to the placental receptor. In helminth infections, antibody isotype patterns are an indicator of the host’s immune regulatory status, pointing to the relative level of IgG4 as a corollary of chronic parasite carriage favored by regulatory cytokines. Pathology of chronic helminth infections are characterized by elevated IL-10 and TGFβ that mediate IgG4 production by B cells (23–25). In the context of the STOPPAM study, sub-microscopic parasite carriage revealed by PCR-based diagnosis carried out with samples from this cohort (26) shows that the women who have elevated IgG4 levels are consistently those with low-density (sub-microscopic) parasite carriage at 4 different time points during pregnancy, strongly suggestive of persisting chronic infections. As we have always found, in all our studies, that IL-10 is elevated in the presence of *P. falciparum* infection, whether peripheral (27) or placental (14), we conclude that the conditions therefore likely favored IgG4 production. Further, data obtained using a ICAM-1 binding DBL-β domain protein showed no association with placental infection. This data suggest that our observation with PAMVAC antigen was pregnancy specific and not donor specific. It is also known that the IgG4 titers fall rapidly following curative drug treatment as compared to other subclasses (28). In the context of intermittent preventive treatment administered to pregnant women it is possible that this has contributed to disrupting the association with active infections detected by microscopy in this study. However, the plasma levels of IgG1 and IgG2 subclasses appeared here to represent markers of infection. The observations from this study thus further underline the complex mechanisms underlying antibody responses, the properties of which can both mark the presence of an infection and the success of its control. While this study clearly highlights the importance of the IgG3 subclass in the acquired protection against placental malaria, it also demonstrates for the first time the role that IgG4 could play in this protective mechanism.

One of the limitations of this study is based on the lack of functional data to demonstrate the particular properties of the various purified IgG subclasses. This study opens up on the need for some perspective to confirm the observations. The fact of having used other recombinant antigens reproducing the minimal binding domains of other PfEMP1 (ICAM-1 MBD) and for which we did not observe the same association suggests that the data of this work are of obvious interest. The other limitation is the absence of similar data on the full-length VAR2CSA or other epitopes of the VAR2CSA in this study which would have allowed verify if this association is specific to VAR2CSA in its entirety or just a portion.

In conclusion, the association of IgG3 subclass antibody levels with the risk of low birth weight and that of IgG4 to that of placental infections at delivery, strongly suggest mechanisms of protection mediated by antibody responses against VAR2CSA that rely on different IgG subclasses. This observation further suggests that PM vaccines should ideally target the elicitation of both IgG3 and IgG4 to achieve optimal protection.

## Data Availability Statement

The raw data supporting the conclusions of this article will be made available by the authors, without undue reservation.

## Ethics Statement

The studies involving human participants were reviewed and approved by the Comité Consultatif de Déontologie et d’Éthique of IRD in France and the Comité d’Éthique de la Faculté des Science de la Santé, Université d’Abomey Calavi, in Benin. The patients/participants provided their written informed consent to participate in this study.

## Author Contributions

NTN, PD and MA contributed to the design of the STOPPAM project. FV and NF collected the samples. BT and GE carried out all the lab experiments. MN provided us with the PAMVAC antigen. Td’A performed the statistical analysis. BT and NTN wrote the MS. AL reviewed the MS. All authors contributed to the article and approved the submitted version.

## Funding

BT was supported by a joint ARTS PhD fellowship from IRD and SCAC fellowship from the French Embassy in Ghana.

## Conflict of Interest

The authors declare that the research was conducted in the absence of any commercial or financial relationships that could be construed as a potential conflict of interest.
